# Evolution of the electrical double layer with electrolyte concentration probed by second harmonic scattering†

**DOI:** 10.1039/d3fd00036b

**Published:** 2023-07-17

**Authors:** Bingxin Chu, Denys Biriukov, Marie Bischoff, Milan Předota, Sylvie Roke, Arianna Marchioro

**Affiliations:** a Laboratory for Fundamental BioPhotonics (LBP), Institute of Bioengineering (IBI), Institute of Materials Science (IMX), School of Engineering (STI), École Polytechnique Fédérale de Lausanne (EPFL) CH-1015 Lausanne Switzerland arianna.marchioro@epfl.ch; b Institute of Organic Chemistry and Biochemistry, Czech Academy of Sciences Flemingovo Nám. 2 16610 Prague 6 Czech Republic; c Department of Physics, Faculty of Science, University of South Bohemia Branišovská 1760 370 05 České Budějovice Czech Republic

## Abstract

Investigating the electrical double layer (EDL) structure has been a long-standing challenge and has seen the emergence of several sophisticated techniques able to probe selectively the few molecular layers of a solid/water interface. While a qualitative estimation of the thickness of the EDL can be obtained using simple theoretical models, following experimentally its evolution is not straightforward and can be even more complicated in nano- or microscale systems, particularly when changing the ionic concentration by several orders of magnitude. Here, we bring insight into the structure of the EDL of SiO_2_ nanoparticle suspensions and its evolution with increasing ionic concentration using angle-resolved second harmonic scattering (AR-SHS). Below millimolar salt concentrations, we can successively characterize inner-sphere adsorption, diffuse layer formation, and outer-sphere adsorption. Moreover, we show for the first time that, by appropriately selecting the nanoparticle size, it is possible to retrieve information also in the millimolar range. There, we observe a decrease in the magnitude of the surface potential corresponding to a compression in the EDL thickness, which agrees with the results of several other electroanalytical and optical techniques. Molecular dynamics simulations suggest that the EDL compression mainly results from the diffuse layer compression rather than outer-sphere ions (Stern plane) moving closer to the surface.

## Abstract 2

We follow the evolution of the electrical double layer of colloidal SiO_2_ particles from the micromolar to the millimolar regime using nonlinear optical techniques and evidence a diffuse layer compression.

## Graphical abstract

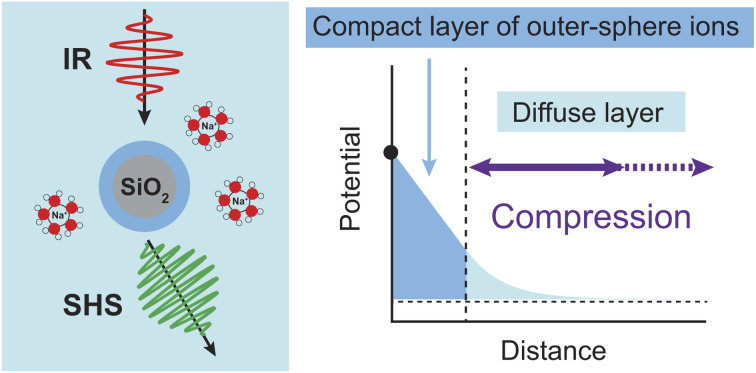

## 1 Introduction

The build-up of surface charge and distribution of ions at solid/water interfaces play a central role in various processes in chemistry, electrochemistry, geology, biology, and technological applications. When sufficiently close to a charged surface, ions and dipolar molecules order and change their orientation to oppose the effect of the surface electric field. Theoretical models of the solid/electrolyte interface, or electrical double layer (EDL), had been established already at the beginning of the 20th century and could, to a certain extent, explain or predict the interfacial behavior. However, even common phenomena such as ion specificity or surface charge inversion cannot be directly predicted with classical double-layer models.^1,2^ For this reason, various sophisticated techniques have been proposed in recent years to complement the existing toolbox of electroanalytical and spectroscopic techniques available to study electrified interfaces. Those techniques include high-resolution atomic force microscopy which, *e.g.*, was able to reveal with atomic-level precision the ordered adsorption of mono- and divalent ions at the surface of gibbsite/silica surfaces,^3^ and ambient pressure X-ray photoelectron spectroscopy, which was capable of discerning the shape of the EDL profile.^4^ One aspect frequently discussed is the actual “thickness” of the EDL and its dependence on the nature or concentration of the aqueous electrolyte solution. The classical description of the interfacial region relies on the Poisson–Boltzmann theory of the distribution of point charges, where the thickness of the EDL is characterized by the Debye length. The Debye length is a practical approximation, although it does not take into account ion specificity and/or the possibility of ion adsorption at the surface. The subject of the EDL thickness has been extensively addressed on the theoretical level,^5–10^ however, much less has been done on the experimental side. The same ambient XPS study cited above^4^ provided an estimation for the EDL thickness as a function of ionic concentration, while for nanoparticles dispersed in solution, to our knowledge only liquid-jet XPS experiments reported on the EDL thickness.^11^ In this work, we show that angle-resolved second harmonic scattering (AR-SHS) can be used as a probe of the EDL evolution with increasing ionic concentration around colloidal particles dispersed in solution, from the lowest possible electrolyte concentrations up to millimolar concentrations. SHS is based on the same principles of second harmonic generation – the better-known optical technique applied to planar interfaces – but it can be applied to any scattering object dispersed in solution. The SHS signal arises from the breaking of the centrosymmetry in the system, that is, the interface between the scattering particles and the aqueous solution for centrosymmetric particles in water. There, SHS is sensitive to the local order in the system, which, in the absence of second harmonic generating dyes, mainly originates from water dipoles aligned near the scatterer/water interface by means of chemical interactions with the surface.^12,13^ A second contribution to the local order can manifest when a surface electrostatic field is present in the interfacial region, which is a common situation in the case of particles dispersed in solution. In this second case, water dipoles further away from the interface can be aligned by electrostatic interactions.^14,15^ These two contributions to the local order result in the total SHS signal and are contained in two quantities that can be extracted from AR-SHS data using a model developed in the Roke group:^16^ the surface potential *Φ*_0_ and the second-order surface susceptibility *χ*^(2)^_s,2_. By appropriately selecting the particle diameter, we show that *Φ*_0_ and *χ*^(2)^_s,2_ trends as a function of ionic concentration allow the identifying of different regimes corresponding to inner-sphere adsorption, diffuse layer formation, outer-sphere adsorption, and compression of the EDL. For the last phenomenon, molecular dynamics simulations indicate that a thinner diffuse layer, rather than a thinner layer of outer-sphere ions, is involved. We compare our results to state-of-the-art techniques able to probe the EDL and discuss their complementarity to better understand the EDL and its properties.

## 2 Experimental section

### 2.1 Chemicals

Sodium chloride (NaCl, >99.999%, Sigma-Aldrich) was used as received. SiO_2_ colloids of different nominal sizes (100, 200 and 300 nm) were purchased from Polysciences/Bangs Laboratories, Inc. 100 nm and 200 nm diameter SiO_2_ nanoparticles were received in aqueous solutions (6.0% w/w and 10.26% w/w, respectively), while 300 nm diameter SiO_2_ nanoparticles were received as a powder. The measured hydrodynamic diameter was found to be in some cases slightly different from the nominal size (see Section 2.3).

### 2.2 Sample preparation

Ultrapure water (Milli-Q, Millipore, Inc., electrical resistance of 18.2 MΩ × cm) was used to prepare all the samples. For 100 nm SiO_2_ samples, the purchased stock solution was sonicated for 10 min (35 kHz, 400 W, Bandelin) and vortexed for 2 min prior to usage. Then the stock was diluted in water to 0.5% w/w, sonicated again for 3 min and vortexed for 2 min. To remove residual ions from the synthetic procedure, the 0.5% w/w solution was centrifuged for 10 min at 7800 rpm (5430R, Eppendorf). Then, 9 mL of the supernatant was removed and the pellet was resuspended in the same volume of MilliQ water by vortexing and ultrasonication for 3–5 min. The conductivity of the washed solution was measured using a conductivity meter calibrated with the appropriate buffer solutions (HI 76312 conductivity electrode, Hanna Instruments) to ensure that the initial ionic strength was as low as possible (the residual ionic strength originates from the presence of residual ions from the synthetic process and the presence of HCO_3_^−^ ions from the dissolution of atmospheric CO_2_). The SiO_2_ particle suspensions were further diluted to 0.05% w/w solutions containing the desired amount of NaCl, corresponding to approximately 2.3 × 10^11^ particles per mL and to a total surface area of ∼1.2 × 10^−2^ m^2^ mL^−1^, using for these estimations the hydrodynamic diameter measured by dynamic light scattering (see Section 2.3). The ionic strength of the solutions was adjusted with 0.01 M solutions of NaCl. Corresponding water references at the same ionic strength were prepared for each SiO_2_ sample. All preparation steps and measurements were performed at room temperature.

The 200 and 300 nm samples were prepared following a similar procedure as the 100 nm ones. The particle concentration of both the 200 and 300 nm samples was adjusted to ensure a similar total surface area as the 100 nm sample. For 200 nm samples, the stock solution was first diluted to 0.7% w/w and then to 0.07% w/w samples containing the desired amount of NaCl. The corresponding particle concentration and total surface area are approximately 1.3 × 10^11^ particles per mL and ∼1.2 × 10^−2^ m^2^ mL^−1^. For 300 nm particles, 100 mg of SiO_2_ nanoparticles were first dispersed in 1 mL of ultrapure water, sonicated for 15 min, and then diluted to 10 mL with ultrapure water to prepare a 1% w/w solution. After being centrifuged and sonicated, particles were further diluted to 0.1% w/w, corresponding to a particle concentration of 3.3 × 10^10^ particles per mL and a total surface area ∼1.0 × 10^−2^ m^2^ mL^−1^. The ionic strength of the solutions was adjusted with 0.01 M and 1 M solutions of NaCl. A slightly lower surface area was used in the case of the 300 nm particles with respect to the 100 and 200 nm samples in order to remain in the linear range of the SH signal. Indeed, for each sample size, the linearity of the SH scattering signal with particle concentration, indicating the absence of multiple scattering events, was ensured in separate dynamic light scattering experiments (*i.e.*, the range of linearity of the scattered signal as a function of particle concentration was determined). A particle concentration of 0.05% w/w for 100 nm particles, 0.07% w/w for 200 nm particles and 0.1% w/w for 300 nm particles fulfilled both the criteria of similar surface area as well as the absence of multiple scattering events.

### 2.3 Sample characterization

For each sample, the particle size distribution was measured by dynamic light scattering (DLS) and the zeta potential (*ζ*) was measured by electrophoresis. The mean hydrodynamic diameters measured by DLS (Zetasizer Ultra, Malvern) for 100, 200, and 300 nm SiO_2_ samples were ∼130, 177, and 310 nm, respectively (see Tables in ESI†), with a narrow distribution (for most samples, the polydispersity index (PDI) was <0.1). Similarly to our previous works,^17,18^*ζ* was calculated from electrophoretic mobilities using Ohshima’s approximation.^19^ The conductivity of the solution was obtained using the conductivity meter mentioned in Section 2.2. From the measured conductivity *σ*, the average ionic strength, which is equivalent to the ionic concentration for monovalent ions, can be calculated as shown in our previous studies.^13,20^

### 2.4 AR-SHS measurements

Second harmonic scattering measurements were performed on the same AR-SHS setup as described in ref. 21. In an AR-SHS measurement, the 1032 nm fundamental beam is generated by a mode-locked Yb:KGW laser (Pharos-SP, Light Conversion) with a 190 fs pulse duration and a 200 kHz repetition rate. The polarization of the fundamental beam is controlled by a Glan–Taylor polarizer (GT10-B, Thorlabs) and a zero-order half-wave plate (WPH05M-1030) to be either horizontal (P, parallel to the scattering plane) or vertical (S, perpendicular to the scattering plane). The beam is further filtered using a long-pass filter (FEL0750, Thorlabs) and then focused into the cylindrical glass cuvette containing the sample (LS instruments, 4.2 mm inner diameter) with a plano-convex lens (*f* = 7.5 cm). The beam power at the sample was set to 62 mW, corresponding to a fluence at the focus of ∼3.4 mJ cm^−2^. The 516 nm SH signal is scattered from the SiO_2_/water interface, collected and collimated with a planoconvex lens (*f* = 5 cm), polarization-analyzed using a Glan–Taylor polarizer (GT10-A, Thorlabs) and filtered by a 516 ± 10 nm filter (CT516/10bp, Chroma) before being focused into a gated photomultiplier tube (H7422P-40, Hamamatsu). The acceptance angle was set to 3.4° for scattering patterns. Patterns were obtained in steps of 5° from *θ* = −90° to *θ* = 90° with 0° being the forward direction of the fundamental beam. The signal was acquired with a gated photon counter (SR400, Stanford Research Instruments) with an acquisition time of 1.5 s/*θ*. Each data point was recorded as an average of 20 measurements. Two polarization combinations (PPP and PSS) are measured for each sample. The first letter corresponds to the polarization of the outcoming second harmonic beam, while the two last letters correspond to the polarization of the incoming excitation beam. To correct for incoherent hyper-Rayleigh scattering (HRS) from the solvent phase, both the SHS response from the sample solution *I*_PXX,sample_(*θ*) and the HRS response from a solution *I*_PXX,solution_(*θ*) of identical ionic strength but without nanoparticles need to be collected. The HRS is subtracted from the SHS signal of the sample and the obtained difference is then normalized to the isotropic SSS signal of pure water to correct for day-to-day differences in the beam profile. We give here the normalized signal of the sample *S*(*θ*)_PPP_ for AR-SHS in the PPP polarization combination:1
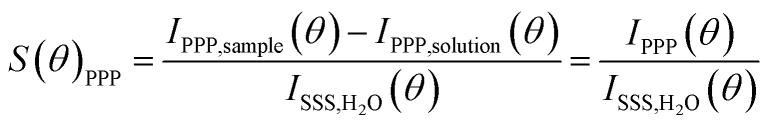


The normalization procedure was applied in the same way for AR-SHS measured in the PSS polarization combination. The normalized patterns were then fitted using the AR-SHS model previously derived^16^ in order to extract the surface potential *Φ*_0_ and the second-order surface susceptibility *χ*^(2)^_s,2_.^22–24^ For a summary of the key points of the model as well as the fitting equations, we refer the reader to the theoretical background section in ref. 24.

### 2.5 Molecular dynamics simulations

Molecular dynamics simulations were carried out using similar simulation protocols and force field parameters as in our earlier work.^13,17,20,25^ SiO_2_ was modeled as a macroscopically flat crystal (101) quartz surface,^25^ which is a faithful representation of the metal oxide/water interface of large SiO_2_ nanoparticles. To mimic the experimental pH environment, a surface charge density of −0.03 C m^−2^ was modeled, which is equivalent to 3.125% of deprotonated silanols from the total amount.^25^ A simulation cell consisted of two 5.5 × 3.928 nm SiO_2_ five-Si-layer slabs separated by a ∼16 nm thick aqueous solution containing 11 300 water molecules and varying amounts of Na^+^ and Cl^−^ ions. In addition to 8 Na^+^ counter-ions always present in the system to compensate the surface charge of both surfaces, additional zero, 8, 16, 24, or 32 NaCl ion pairs were added to model the range of different bulk concentrations from ∼7 to ∼170 mM. Note that in the case of the system with only Na^+^ ions, half of the bulk concentration of sodium is reported as the bulk salt concentration since no chloride ions are present in the system. The water was modeled as rigid SPC/E,^26^ and the force field parameters for ions were taken from the literature.^27^ The models for both the SiO_2_ surface and ions employ the so-called scaled charges that account for electronic polarization in nonpolarizable force fields.^28,29^ The reported MD data are averages over the two interfaces present in the simulation cell. The simulations were carried out in a canonical NVT ensemble and were 1 μs long, with the first 100 ns being considered as equilibration and disregarded from the analysis. The temperature of 298.15 K was maintained using the Nosé–Hoover thermostat^30,31^ with a coupling time of 1 ps. The cut-off for electrostatic and van der Waals interactions was set to 1.2 nm. The long-range electrostatic contribution was calculated using the particle mesh Ewald method^32^ with the correction for slab geometry.^33^ The water geometry was constrained using the SETTLE algorithm.^34^ All other covalent bonds involving hydrogens were constrained using the LINCS algorithm.^35^ All simulations were performed in Gromacs 2021.6 (ref. 36) and the post-processing was carried out using Gromacs built-in tools.

## 3 Results and discussion

### 3.1 Surface potential for 100, 200 and 300 nm SiO_2_ particles

**Fig. 1:**
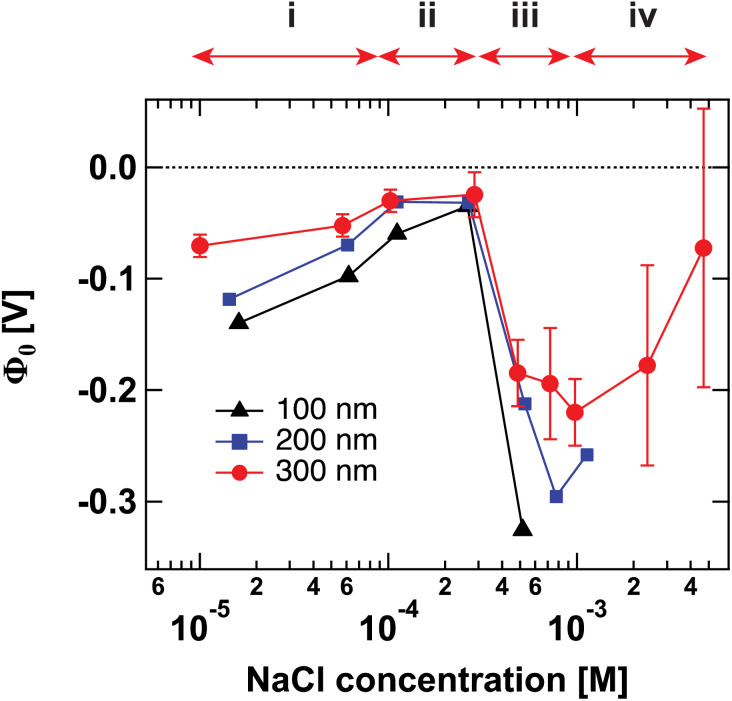
Surface potential *Φ*_0_ for 100 nm (black triangles), 200 nm (blue squares), and 300 nm-diameter amorphous SiO_2_ particles (red circles) as a function of NaCl concentration. The values of *Φ*_0_ are extracted by fitting the patterns shown in Fig. S1† using the method summarized in ref. 24. The arrows indicate the different regions discussed in the text: (i) inner-sphere adsorption, (ii) diffuse layer formation, (iii) outer-sphere adsorption, (iv) compression of the diffuse layer. Region (iv) can only be evidenced with larger-size particles.

Fig. 1 shows the surface potential *Φ*_0_ for SiO_2_ particles of different sizes in NaCl solutions of varying ionic strength. *Φ*_0_ has been extracted from the fits of the AR-SHS patterns (see ESI†) following the procedure detailed in ref. 22–24. We designate *Φ*_0_ as the difference between the potential of the surface and the potential of the bulk electrolyte. It is important to note that *Φ*_0_ is extracted from the AR-SHS data without implying a model for the charge distribution close to the interface, and as such, it gives direct information on the interfacial electrostatics.

Fig. 1 has been divided into four different regions indicated by the red arrows: (i), (ii), (iii), and (iv). The behavior of *Φ*_0_ and *χ*^(2)^_s,2_ in the first three regions, together with a comparison to zeta potential *ζ*, has been discussed in detail in our previous works on TiO_2_ and SiO_2_ nanoparticles.^17,20^ We will briefly describe the physical meaning of each region and then concentrate on Region (iv).

#### 3.1.1 Region (i)

Region (i) spans from the lowest reported concentrations up to ∼100 μM for all sizes and represents the region where |*Φ*_0_| initially decreases. We have previously assigned this decrease in the magnitude of *Φ*_0_ to cations being adsorbed at the slightly deprotonated particle surface. For SiO_2_ particles in neutral aqueous solutions, one can expect a ∼3% deprotonation as estimated in ref. 17. At these deprotonated silanol sites, cations can adsorb as inner-sphere complexes (*i.e.*, as partially dehydrated ions), which therefore causes screening of the surface electrostatic field penetrating into the solution.^20^

#### 3.1.2 Region (ii)

Region (ii) spans from ∼100 μM to ∼300 μM. Here, |*Φ*_0_| reaches its lowest absolute value for all three sizes and does not change significantly with increasing ionic strength up to 300 μM. Because favorable deprotonated surface sites are expected to be occupied already at the lowest salt concentrations, we have attributed the behavior of |*Φ*_0_| in Region (ii) to the formation of a diffuse layer, designated here as a layer of cations with their intact hydration shell distributed in solution and similar to the traditional picture presented by the Gouy–Chapman model, where the characteristic thickness of the charge imbalanced diffuse layer is given by the Debye length *κ*^−1^. The increasing amount of ions leads to a decrease of the Debye length (*i.e.*, they increase the electrical screening of the surface charge) and consequently decreases the magnitude of the surface potential |*Φ*_0_|. For the salt concentrations we use, we do not expect a significant increase in inner-sphere adsorption, as this would result in colloidal instability and particles precipitating out of the solution, which we do not observe in this range of ionic strengths. Note that at this stage, inner-sphere adsorption and the decrease in *κ*^−1^ cannot be easily deconvoluted experimentally, as both phenomena contribute to the decrease of |*Φ*_0_| and might result in a partial overlap of Regions (i) and (ii).

#### 3.1.3 Region (iii)

Region (iii) comprises data points at ∼250 μM and 500 μM for 100 nm particles, from ∼250 μM to 750 μM for 200 nm particles, and from ∼250 μM to 1 mM for 300 nm particles. Here, the steep increase in |*Φ*_0_| for all particle sizes was attributed to the gradual approach of outer-sphere ions forming a compact layer of hydrated ions close to the particle surface. The presence of a large number of outer-sphere ions may induce a strong electric field between the negatively charged deprotonated silanols and the positively charged sodium cations. While our data were retrieved without making assumptions about the electrostatic structure of the interface, our conclusions point towards the formation of a layer similar to the Stern layer postulated by the Gouy–Chapman–Stern model. In such a model, the steep potential increase at the surface is associated with the formation of a parallel plate capacitor where the potential drop in the capacitor is given by:2
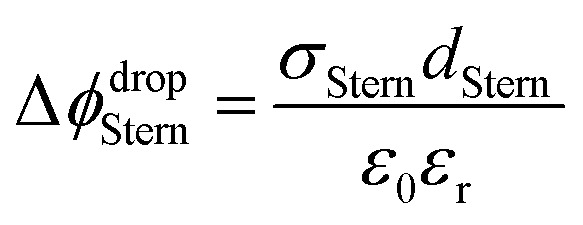
where *σ*_Stern_ is the surface charge density involved in the capacitor, which might be only a fraction of the total surface charge density *σ*_0_, or more accurately, a fraction of the surface charge density effectively perceived by the counterions *σ*_0_ − *σ*_inner-sphere_ (due to the presence of inner-sphere adsorbed ions). *d*_Stern_ is the distance between the capacitor’s plates, *ε*_0_ the vacuum permittivity, and *ε*_r_ is the permittivity of this part of the solid/water interface, which can be very different from the permittivity in the diffuse layer. The permittivity of water molecules close to the surface can be reduced due to preferential alignment resulting from specific interactions of water molecules with surface groups, where the preferential orientation is dictated by the surface charge.^37^ This effect can be further augmented by the presence of ions which are known to reduce the permittivity^38^ – particularly taking into account that the local concentration of ions at the interface can be significantly higher than the bulk concentration.^39^

With increasing salt concentration, a larger fraction of counterions is expected to participate in the Stern layer, and *Φ*_0_ can be expressed as the sum of two contributions:

3 *Φ*_0_ = Δ*ϕ*^drop^_Stern_ + Δ*ϕ*^drop^_diffuse_

where in Region (iii) the increase in salt concentration makes the magnitude of the first term larger, while the reduction of the magnitude of the second term is less significant, therefore explaining the increase in |*Φ*_0_|. We note that the spherical capacitor equation would be more appropriate in the case of colloidal particles. However, the latter reduces to the parallel plate capacitor equation in the limit where the Stern layer thickness is much smaller than the particle radius.

#### 3.1.4 Region (iv)

We had previously hypothesized that, if our interpretation of the first three regions was correct, a further increase in ionic strength beyond Region (iii) keeping a constant surface charge density should lead to a compression of the compact layer of hydrated ions, or outer-sphere ions getting closer to the surface.^17^ In the idealized capacitor model, a compression would result in a decrease in *d*_Stern_ and lead to a decrease in Δ*ϕ*^drop^_Stern_, consequently also decreasing the magnitude of *Φ*_0_. While the proposal of a “thinner” Stern layer with increasing concentration has often been put forward in the literature, few experimental techniques would be able to observe such a phenomenon on a molecular scale. Recently, liquid-jet X-ray spectroscopy of SiO_2_ particles in salt concentrations > 10 mM supported this hypothesis.^11^ Another possible simplified picture would imply the gradual formation of a more compact diffuse layer, *i.e.*, more ions in the diffuse layer participating in screening the remaining surface charge density (*σ*_0_ − *σ*_inner-sphere_ − *σ*_Stern_) and a consequent decrease in the Debye length. In other words, a screening in the diffuse layer would correspond to a compression of the diffuse layer (as opposed to the compression of the Stern layer mentioned above). Previously, our data collected in the micromolar regime for 100 nm TiO_2_ and SiO_2_ particles could not directly investigate phenomena occurring in Region (iv). Because of the low signal-to-noise (S/N) of our experiment in the mM regime, we were not able to retrieve reliable surface potential values at higher ionic strengths, where the compression of either the Stern layer or the diffuse layer could be expected.

Recently, we were able to show that the interferences between surface and electrostatic contributions, which are responsible for the total AR-SHS signal intensity, could not only be modulated by ionic strength but were additionally highly dependent on the interplay between particle size and the material’s specific surface properties.^24^ For SiO_2_ particles in neutral aqueous solutions, the difference in AR-SHS intensity between 100 and 300 nm particles of comparable total surface area can be quite significant. A direct consequence of this finding is that larger particle sizes may allow us to investigate regions at higher ionic strength than previously reported for AR-SHS. For 300 nm SiO_2_ particles, the signal intensity above 1 mM was sufficient to allow us to record AR-SHS patterns with adequate S/N and obtain more reliable fitting results than in the case of 100 and 200 nm particles (see patterns in ESI†). Despite error bars being still relatively large at concentrations > 1 mM, the decreasing trend in |*Φ*_0_| was confirmed on different batches of 300 nm SiO_2_ particles. The results of such measurements constitute the experimental observation of Region (iv). As visible in Fig. 1, |*Φ*_0_| decreases at concentrations > 1 mM, in agreement with the experimental results from Brown *et al.* performed at concentrations > 10 mM.^11^ Our experiments therefore clearly demonstrate that past a specific threshold, additional ions introduced to the system must contribute to a decrease in |*Φ*_0_|. However, AR-SHS cannot directly distinguish between the hypothesis of a “thinner” Stern layer or a more compact diffuse layer. In order to provide insights into the mechanism at the root of the decrease of |*Φ*_0_| and a clearer picture of ions in different hydration environments, we turned to molecular dynamics (MD) simulations.

Although the range of investigated concentrations by MD simulations (from ∼7 mM to 170 mM) is higher than the experimental one, we still can describe the properties of the SiO_2_/water interface assuming that MD simulations also operate in the Region (iv) as well as the SHS experiments at concentrations > 1 mM. The main argument supporting this assumption is that increasing concentration in the millimolar range mainly implies adding more mobile charged carriers without significantly affecting the ionic distribution among available adsorption sites and their position with respect to the surface. As discussed below, this concentration trend is the primary reason for the changes in surface potential and surface susceptibility observed experimentally in Region (iv).

**Fig. 2:**
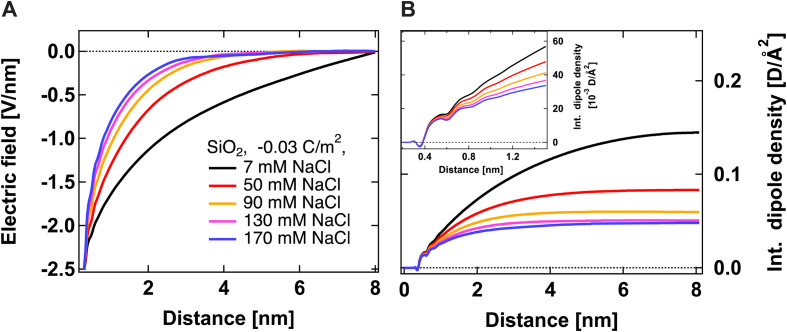
Electric field *E*_*z*_ (A) and integrated dipole density (B) as a function of *z*-distance from the surface from simulations with a constant surface charge density of −0.03 C m^−2^ but at different ionic concentrations. The electric field is calculated only from ionic and SiO_2_ charges, *i.e.*, without the contribution from water molecules. The dipole density is the product of the number density of water molecules, the cosine of the angle between the surface normal and water dipole, and the dipole moment of the SPC/E water model equal to 2.35 D.

**Fig. 3:**
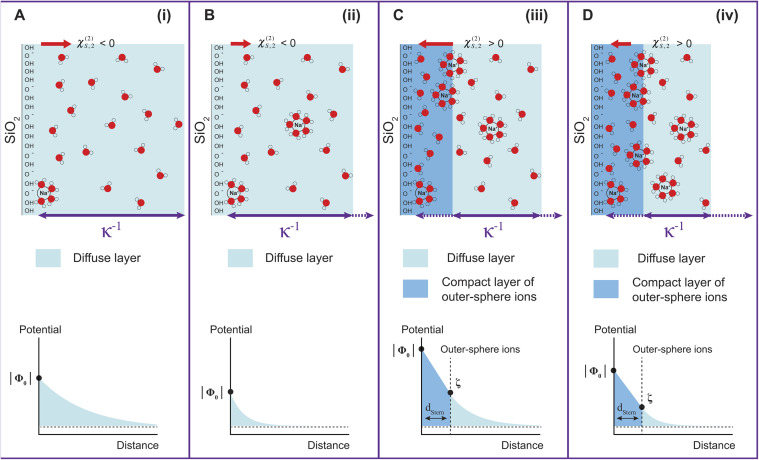
Schematic representation of the gradual evolution of the EDL with increasing ionic concentration (top). The decay of the surface potential magnitude is schematically shown on the bottom part. (A) Very low ionic strength, corresponding to Region (i); (B) low ionic strength corresponding to Region (ii); (C) higher ionic strength corresponding to Region (iii), where a compact layer of outer-sphere ions is formed. This is analogous to the Stern layer in the Gouy–Chapman–Stern model; (D) ionic strength in the millimolar regime, corresponding to Region (iv), where the compression of the diffuse layer occurs with increasing ionic strength. *κ*^−1^ represents the Debye length, or the distance over which the ion distribution differs from the bulk electrolyte.

Fig. 2A shows the electric field decay as a function of *z*-distance from the surface from simulations with a constant surface charge density of −0.03 C m^−2^ (corresponding approximately to pH = 7) at different NaCl concentrations. We observe more efficient surface charge screening with increasing ionic concentration, as indicated by the steeper decay of the electric field. Note that the decay observable up to ∼8 nm at the lowest concentration is somewhat a pathological case since we have only sodium counter-ions in the system, *i.e.*, the decay expands as far as the system box size allows. At the same time, we do not observe changes in the position of adsorbed cations in close vicinity of the surface under any conditions, see Fig. S2,†*i.e.*, we do not observe the geometric compression of the Stern plane (or outer-sphere ions). Our results instead suggest that the decrease in the surface potential is caused by the additional ions screening the remaining electric field and shrinking the diffuse layer (see the scheme in Fig. 3).

Fig. 2B shows the integrated dipole density as a function of *z*-distance from the surface from simulations with a constant surface charge density of −0.03 C m^−2^ at different NaCl concentrations. As described in our previous work,^13^ this quantity corresponds to the integral of the dipole density, given by the product of the number density of water molecules, water dipole orientation (the cosine of the angle between the water dipole and surface normal), and the dipole moment of the SPC/E water model (2.35 D). Positive values indicate a preferred water orientation with hydrogens facing the surface. In our case, the dominant contribution to the SHS signal intensity is due to water dipoles aligned near the scatterer/water interface (surface response) and further away from the surface by electrostatic interactions (electrostatic response) (see ref. 24 for detailed explanations). As such, the integrated dipole density is a measure of the build-up of the total SHS intensity: the SHS intensity is proportional to the square of the running integral of the dipole density.^13^ One can see that the integrated dipole density decreases with increasing ionic concentration, and at higher NaCl concentrations, a plateau is reached at distances closer to the surface. This plateau implies that the electric field generated by the surface is fully screened, *i.e.*, the bulk behavior of water is recovered at larger distances. Fig. 2B therefore clearly indicates the saturation of water alignment, and that fewer water molecules are aligned with increasing NaCl concentration, therefore corroborating the results of Fig. 2A. The inset of Fig. 2B shows the integrated dipole density within the first nanometer away from the interface, which can be linked to the second quantity that we infer from our AR-SHS, the surface susceptibility *χ*^(2)^_s,2_ discussed in the next section.

Finally, coming back to the experimental results, it is interesting to compare *Φ*_0_ with the values of zeta potential (*ζ*) calculated from electrophoretic mobilities, which is traditionally interpreted as the potential at the slipping plane.^40^ While *ζ* values do not display large variations with increasing ionic strength as compared to *Φ*_0_ (see Fig. S3†), it is clear that for the 300 nm SiO_2_ particles, |*ζ*| has decreased by half between 500 μM and 5 mM. A decrease of |*ζ*| from 0.01 to 0.1 M NaCl has also been observed by Brown *et al.* for their 9 nm SiO_2_ particles.^11^ One assumption commonly made is that the slipping plane is located at the very start of the diffuse layer, or in other words, the Stern plane and the slipping plane overlap. In this case, |*ζ*| = Δ*ϕ*^drop^_diffuse_ (eqn (3)) and one could use the low-potential approximation for a plate-like particle, 
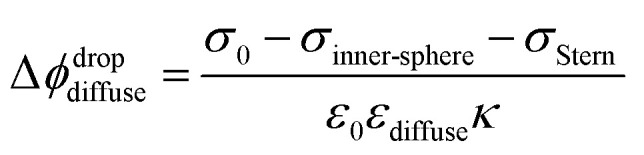
, with *ε*_diffuse_ being the permittivity of the diffuse layer.^41^ Therefore, one can see from this simple equation that a decrease in *κ*^−1^ with increasing ionic concentration would imply a smaller Δ*ϕ*^drop^_diffuse_ and a smaller |*ζ*|, which is what we observe experimentally. A decreasing Δ*ϕ*^drop^_diffuse_ with increasing ionic concentration is also in line with the compression of the diffuse layer. It is interesting to note the fact that the values of *Φ*_0_ and *ζ* differ significantly in Regions (iii) and (iv). The fact that their concentration dependence is even opposite in Region (iii) further supports our observation that the most significant changes to the EDL in Region (iii) occur at the compact layer (see Fig. 3), which is responsible for the first term in eqn (3). That term contributes to the total value of *Φ*_0_, but should have limited direct impact on *ζ*, which is rather associated with the second term in eqn (3) arising from the diffuse layer. Changes of the diffuse layer should therefore have similar effects on both *Φ*_0_ and *ζ*, which is the case in Region (iv), while in Region (iii) we observe opposite trends, attributed to changes of the compact layer. The relation of *Φ*_0_ and *ζ* is more complex though: while *Φ*_0_ depends purely on the electrostatics, *ζ* results from a gentle interplay of electrostatics and dynamics at the interface.^40,42^

### 3.2 Second-order surface susceptibility for 100, 200 and 300 nm SiO_2_ particles

**Fig. 4:**
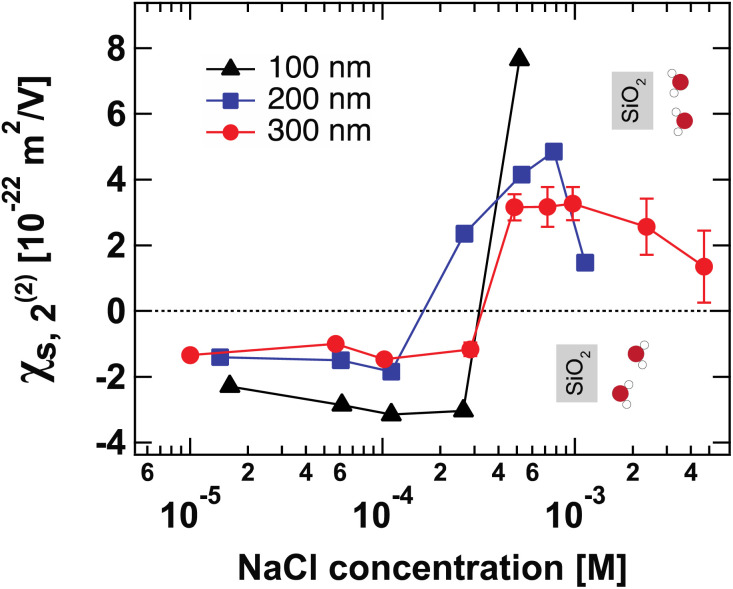
Surface susceptibility *χ*^(2)^_s,2_ for 100 nm (black triangles), 200 nm (blue squares), and 300 nm-diameter amorphous SiO_2_ particles (red circles) as a function of NaCl concentration. The values of *χ*^(2)^_s,2_ are extracted by fitting the patterns shown in Fig. S1† using the method summarized in ref. 24. *χ*^(2)^_s,2_ reflects the average water orientation in the very first molecular layers adjacent to the particle surface. The orientations of water molecules with respect to the SiO_2_ surface giving rise to negative and positive values of *χ*^(2)^_s,2_ are indicated.

Fig. 4 shows the second-order surface susceptibility *χ*^(2)^_s,2_ for SiO_2_ particles of different sizes in NaCl solutions of varying ionic strength. As in the case of *Φ*_0_, *χ*^(2)^_s,2_ has been extracted from the fits of the AR-SHS patterns following the procedure detailed in ref. 22–24 (see ESI† for patterns). *χ*^(2)^_s,2_ reflects the average water orientation in the very first molecular layers adjacent to the particle surface. By convention, a negative sign of *χ*^(2)^_s,2_ is representative of the net dipole moment of interfacial water molecules pointing away from the surface (a majority of oxygen atoms towards the surface), while a positive sign of *χ*^(2)^_s,2_ is representative of the net dipole moment of interfacial water molecules pointing towards the surface (a majority of hydrogen atoms towards the surface).^13^ Our data in Fig. 4 indicate a similar behavior for all three sizes: *χ*^(2)^_s,2_ first displays negative values at low ionic strengths, then a sign reversal to positive values can be observed at higher ionic strengths. The sign reversal occurs between 250 μM and 500 μM for 100 nm particles, between 100 μM and 250 μM for 200 nm particles, and between ∼250 μM and 500 μM for 300 nm particles. This behavior is expected and in agreement with what was observed previously for both SiO_2_ and TiO_2_ samples.^13,20^ Note that the exact turning point for each size can vary slightly and has been found to be batch-dependent. On a molecular level, our *χ*^(2)^_s,2_ results corroborate the picture obtained by analyzing the trends of *Φ*_0_. At low ionic strength, the EDL consists in a slightly deprotonated silanol surface, where sporadic inner-sphere adsorption occurs and a majority of interfacial water molecules orient with oxygens towards the surface (surface OH⋯OH_2_ bond). At higher ionic strength but for constant surface charge density (no further deprotonation of the SiO_2_ surface), outer-sphere adsorption prevails, inducing a reorganization of interfacial water molecules and a reorientation with a majority of hydrogens towards the surface (oxygen atoms towards the outer-sphere cations). Additionally, because of the interferences between surface and electrostatic contributions detailed above for the surface potential case, we can now explore a region at higher ionic strength for the 300 nm particles. Fig. 4 shows that positive values of *χ*^(2)^_s,2_ reach a maximum close to 1 mM and then decrease > 1 mM. This decrease may be representative of two situations: (1) a decrease in the total number of water molecules in the very first layers close to the surface or (2) a weaker orientation preference of water molecules in the very first layers close to the surface. The inset of Fig. 2B, showing the integrated dipole density between 0.35 and 1.35 nm, where the closest adsorbed-water layers are located,^13^ is representative of the *χ*^(2)^_s,2_ obtained by AR-SHS. One can see that the orientation preference of water molecules very close to the surface is indeed decreasing with increasing NaCl concentration, in agreement with the experimental trends shown in Fig. 4.

### 3.3 Comparison of AR-SHS with other experimental methods

**Fig. 5:**
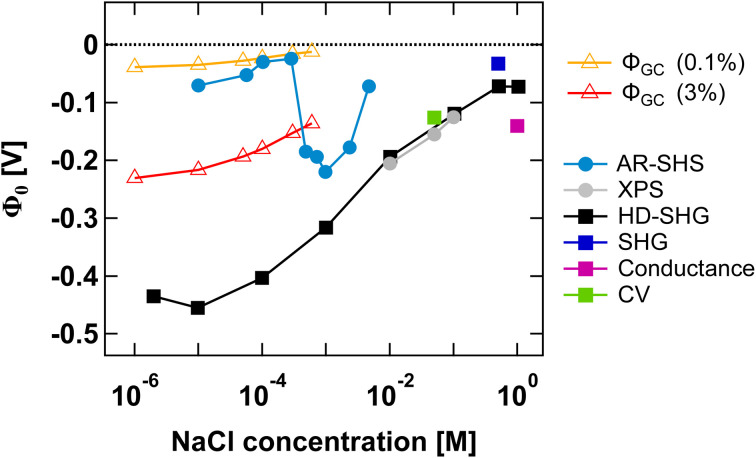
Surface potential *Φ*_0_ as a function of NaCl concentration for the SiO_2_/water interface in neutral pH conditions. *Φ*_0_ values for amorphous 300 nm SiO_2_ particles as determined from AR-SHS in this study are indicated as light blue circular markers. In comparison, we show *Φ*_0_ values taken from the literature and obtained through different techniques: XPS (grey filled circles),^11,43^ heterodyne-SHG (black squares),^44^ SHG (dark blue squares),^14^ conductance method (magenta squares),^45^ and cyclic voltammetry measurements (green squares).^46^ Filled circles are used for SiO_2_ particles, while filled squares indicate planar SiO_2_ surfaces. The orange and red open triangles represent estimates of *Φ*_0_ obtained using surface charge density values reported in the literature for SiO_2_ particles in neutral conditions (corresponding to 0.1% and 3% deprotonation respectively, see the main text) and the Gouy–Chapman (GC) model applied to spherical particles, using the treatment proposed by Ohshima.^41^ The region between the orange and red lines therefore represents a range of possible *Φ*_0_ values for spherical particles in neutral conditions using the GC model and indicates that our AR-SHS results fit within the range of predicted values at low ionic strengths (<∼300 μM), but starts deviating from it for higher ionic strengths (>∼300 μM).

Finally, in Fig. 5 we compare our results to those from other experimental methods able to probe EDL parameters. We have specifically collected *Φ*_0_ values reported in the literature for SiO_2_ in NaCl electrolyte at neutral pH for both colloidal and planar SiO_2_ surfaces. Such comparison is insightful as it illustrates the importance of ionic strength conditions when discussing different methods. To the best of our knowledge, only AR-SHS and liquid jet X-ray spectroscopy (XPS) can measure absolute values of surface potential for colloidal particles in solution. Other indirect methods include potentiometric titrations, where experimentally measured surface charge density can be converted to surface potential using the Gouy–Chapman or Gouy–Chapman–Stern models.

Fig. 5 shows that AR-SHS and liquid jet XPS are complementary as they operate in different ionic strength regimes as we previously discussed.^17^ While AR-SHS cannot be used above a certain ionic strength due to low S/N, liquid jet XPS measurements must be performed in the millimolar regime to reduce photoionization charging effects.^47^ Here, Fig. 5 shows that in the range from 1 mM to 5 mM, |*Φ*_0_| is indeed decreasing, which could not be observed previously when measuring smaller-sized particles in AR-SHS, and in agreement with XPS measurements from 10 mM to 0.1 M. For AR-SHS, we additionally indicate on the graph boundary values corresponding to *Φ*_0_ values retrieved using the Gouy–Chapman model adapted to spherical particles. These calculations use surface charge density values obtained by potentiometric titrations reported in the literature. *Φ*_0_ values shown as red open triangles are calculated with a fixed surface charge density of −22 mC m^−2^ (ref. 48) (corresponding to 3% deprotonation, as discussed in ref. 17), while *Φ*_0_ values plotted as orange open triangles are calculated using a surface charge density of −0.8 mC m^−2^^49^ (corresponding to 0.1% deprotonation). Fig. 5 shows that our AR-SHS results fit within the range of predicted *Φ*_0_ values for spherical particles in neutral conditions and at low ionic strengths using the GC model. At higher ionic strengths (>∼300 μM), |*Φ*_0_| increases, showing the limitations of the GC model.

For planar surfaces, several ways to retrieve surface potential exist. Analogously to our AR-SHS measurements in the scattering configuration, nonlinear optical techniques such as SHG and heterodyne SHG (HD-SHG) also have a *Φ*_0_-dependence, because in both cases the SH response is sensitive to the breaking of the centrosymmetry generated by the surface contribution together with the electrostatic contribution further away in the bulk solution. For planar surfaces, the quantitative extraction of *Φ*_0_ is not straightforward and may require the help of simulations,^50–54^ although methods to do so have been recently proposed.^44,55,56^ We further report values obtained by conductance and cyclic voltammetry. Here, several aspects can be noticed. First, fewer experimental points have been recorded at lower ionic strength, indicating a limitation of some techniques in the low ionic strength regime. HD-SHG can retrieve *Φ*_0_ values in the 10^−6^ to 10^−4^ M range and, in ref. 44, the authors proposed the existence of a new imaginary term in the second-order surface susceptibility of charged interfaces that could contribute to the total SHG response. The difference between the *Φ*_0_ values reported there and those calculated from the Gouy–Chapman theory were attributed to the fact that the GC model considers only coulombic contributions but does not consider dipole or other contributions to the electrostatic potential.^44^ Any additional contribution originating from the surface electric field extending in the bulk material^57^ would in practice be null in the case of nanoparticles, as the symmetry of the system would cause field cancellation to occur between the two sides of the same nanoparticle. Therefore, at low ionic strengths, such reasoning would explain the significant difference between the results obtained for planar and colloidal SiO_2_. Second, at high ionic strengths, the trends reported for *Φ*_0_ agree between colloidal and planar surfaces, although the exact values may differ. In the case of particles, one can expect an increase in the magnitude of the surface charge with a decrease in the particle size for a fixed background salt concentration and pH,^58^ potentially explaining the differences between the 9 nm particles in ref. 11 and 43 and our larger 300 nm diameter particles. Nevertheless, for all data points reported > 1 mM, |*Φ*_0_| appears to decrease with increasing ionic strength, that is, the compression of either the Stern or the diffuse layer must occur. Therefore, all the experimental techniques applied to different systems in this ionic strength region probe the same phenomenon. However, as discussed above, they may not be able to directly distinguish between ions in different hydration environments (outer-sphere ions *vs.* ions farther away in the diffuse layer), prompting the need for molecular dynamics simulations.

## 4 Conclusion

We have shown that AR-SHS brings insight into the structure of the electrical double layer (EDL) of particle suspensions and its evolution with increasing ionic concentration using two quantities: surface potential *Φ*_0_ and surface susceptibility *χ*^(2)^_s,2_. Our previous works, performed in the sub-mM regime, could evidence three main phenomena: inner-sphere adsorption, formation of a diffuse layer, and formation of a compact layer of outer-sphere ions in the vicinity of the surface. Such phenomena in the low ionic strength regime are not readily observable by other experimental techniques. Here we demonstrate that by increasing the size of our nanoparticles from 100 to 300 nm, we are able to retrieve information at even higher ionic strengths (millimolar regime). In this regime, the EDL compression results in a decrease in the surface potential magnitude, which agrees with the results of several other electroanalytical and optical techniques. Our results are supported by molecular dynamics simulations, suggesting that the EDL compression primarily consists of the diffuse layer compression, rather than the outer-sphere ions (Stern plane) moving closer to the surface. In conclusion, AR-SHS is a valuable optical tool to retrieve information on the EDL of colloidal particles and its qualitative evolution with increasing ionic concentration from the micromolar to the millimolar regime. Additionally, selectively focusing on specific phenomena taking place in the EDL may become possible by simply tuning the particle size, and future work should focus on the quantitative determination of the EDL thickness, together with the permittivity of the different layers.

## Conflicts of interest

There are no conflicts of interest to declare.

## Supplementary Material

FD-246-D3FD00036B-s001

## References

[cit1] Lovering K. A., Bertram A. K., Chou K. C. (2016). New Information on the Ion-Identity-Dependent Structure of Stern Layer Revealed by Sum Frequency Generation Vibrational Spectroscopy. J. Phys. Chem. C.

[cit2] Rashwan M., Rehl B., Sthoer A., Darlington A. M., Azam Md. S., Zeng H., Liu Q., Tyrode E., Gibbs J. M. (2020). Structure of the Silica/Divalent Electrolyte Interface: Molecular Insight into Charge Inversion with Increasing pH. J. Phys. Chem. C.

[cit3] Siretanu I., Ebeling D., Andersson M. P., Stipp S. L. S., Philipse A., Stuart M. C., van den Ende D., Mugele F. (2014). Direct observation of ionic structure at solid-liquid interfaces: a deep look into the Stern Layer. Sci. Rep..

[cit4] Favaro M., Jeong B., Ross P. N., Yano J., Hussain Z., Liu Z., Crumlin E. J. (2016). Unravelling the electrochemical double layer by direct probing of the solid/liquid interface. Nat. Commun..

[cit5] Bohinc K., Kralj-Iglič V., Iglič A. (2001). Thickness of electrical double layer. Effect of ion size. Electrochim. Acta.

[cit6] Das S., Chakraborty S., Mitra S. K. (2012). Redefining electrical double layer thickness in narrow confinements: Effect of solvent polarization. Phys. Rev. E.

[cit7] Iván Guerrero-García G., González-Tovar E., Chávez-Páez M., Kłos J., Lamperski S. (2018). Quantifying the thickness of the electrical double layer neutralizing a planar electrode: the capacitive compactness. Phys. Chem. Chem. Phys..

[cit8] Tokmachev M. G., Tikhonov N. A. (2019). Simulation of capacitive deionization accounting the change of Stern layer thickness. J. Math. Chem..

[cit9] Saboorian-Jooybari H., Chen Z. (2019). Calculation of re-defined electrical double layer thickness in symmetrical electrolyte solutions. Results Phys..

[cit10] Keshavarzi E., Abareghi M. (2022). The Effect of Stern Layer Thickness on the Diffuse Capacitance for Size Asymmetric Electrolyte inside the Charged Spherical Cavities by Density Functional Theory. J. Electrochem. Soc..

[cit11] Brown M. A., Goel A., Abbas Z. (2016). Effect of Electrolyte Concentration on the Stern Layer Thickness at a Charged Interface. Angew. Chem..

[cit12] Schürer B., Wunderlich S., Sauerbeck C., Peschel U., Peukert W. (2010). Probing colloidal interfaces by angle-resolved second harmonic light scattering. Phys. Rev. B: Condens. Matter Mater. Phys..

[cit13] Marchioro A., Bischoff M., Lütgebaucks C., Biriukov D., Předota M., Roke S. (2019). Surface Characterization of Colloidal Silica Nanoparticles by Second Harmonic Scattering: Quantifying the Surface Potential and Interfacial Water Order. J. Phys. Chem. C.

[cit14] Ong S., Zhao X., Eisenthal K. B. (1992). Polarization of water molecules at a charged interface: second harmonic studies of the silica/water interface. Chem. Phys. Lett..

[cit15] Zhao X., Ong S., Eisenthal K. B. (1993). Polarization of water molecules at a charged interface. Second harmonic studies of charged monolayers at the air/water interface. Phys. Rev. Lett..

[cit16] Gonella G., Lütgebaucks C., de Beer A. G. F., Roke S. (2016). Second Harmonic and Sum-Frequency Generation from Aqueous Interfaces Is Modulated by Interference. J. Phys. Chem. C.

[cit17] Bischoff M., Biriukov D., Předota M., Marchioro A. (2021). Second Harmonic Scattering Reveals Ion-Specific Effects at the SiO_2_ and TiO_2_ Nanoparticle/Aqueous Interface. J. Phys. Chem. C.

[cit18] Pullanchery S., Kulik S., Okur H. I., de Aguiar H. B., Roke S. (2020). On the stability and necessary electrophoretic mobility of bare oil nanodroplets in water. J. Chem. Phys..

[cit19] Ohshima H. (1994). A Simple Expression for Henry’s Function for the Retardation Effect in Electrophoresis of Spherical Colloidal Particles. J. Colloid Interface Sci..

[cit20] Bischoff M., Biriukov D., Předota M., Roke S., Marchioro A. (2020). Surface Potential and Interfacial Water Order at the Amorphous TiO_2_ Nanoparticle/Aqueous Interface. J. Phys. Chem. C.

[cit21] Gomopoulos N., Lütgebaucks C., Sun Q., Macias-Romero C., Roke S. (2013). Label-free second harmonic and hyper Rayleigh scattering with high efficiency. Opt. Express.

[cit22] Lütgebaucks C., Gonella G., Roke S. (2016). Optical label-free and model-free probe of the surface potential of nanoscale and microscopic objects in aqueous solution. Phys. Rev. B.

[cit23] Bischoff M., Kim N. Y., Joo J. B., Marchioro A. (2022). Water Orientation at the Anatase TiO_2_ Nanoparticle Interface: A Probe of Surface pKa Values. J. Phys. Chem. Lett..

[cit24] Chu B., Marchioro A., Roke S. (2023). Size dependence of second-harmonic scattering from nanoparticles: Disentangling surface and electrostatic contributions. J. Chem. Phys..

[cit25] Kroutil O., Chval Z., Skelton A. A., Předota M. (2015). Computer Simulations of Quartz (101)–Water Interface over a Range of pH Values. J. Phys. Chem. C.

[cit26] Berendsen H. J. C., Grigera J. R., Straatsma T. P. (1987). The missing term in effective pair potentials. J. Phys. Chem..

[cit27] Kohagen M., Mason P. E., Jungwirth P. (2016). Accounting for Electronic Polarization Effects in Aqueous Sodium Chloride *via* Molecular Dynamics Aided by Neutron Scattering. J. Phys. Chem. B.

[cit28] Leontyev I., Stuchebrukhov A. (2011). Accounting for electronic polarization in non-polarizable force fields. Phys. Chem. Chem. Phys..

[cit29] Biriukov D., Kroutil O., Předota M. (2018). Modeling of solid–liquid interfaces using scaled charges: rutile (110) surfaces. Phys. Chem. Chem. Phys..

[cit30] Nosé S. (1984). A molecular dynamics method for simulations in the canonical ensemble. Mol. Phys..

[cit31] Hoover W. G. (1985). Canonical dynamics: Equilibrium phase-space distributions. Phys. Rev. A.

[cit32] Essmann U., Perera L., Berkowitz M. L., Darden T., Lee H., Pedersen L. G. (1995). A smooth particle mesh Ewald method. J. Chem. Phys..

[cit33] Yeh I.-C., Berkowitz M. L. (1999). Ewald summation for systems with slab geometry. J. Chem. Phys..

[cit34] Miyamoto S., Kollman P. A. (1992). Settle: An analytical version of the SHAKE and RATTLE algorithm for rigid water models. J. Comput. Chem..

[cit35] Hess B., LINCS P. (2008). A Parallel Linear Constraint Solver for Molecular Simulation. J. Chem. Theory Comput..

[cit36] Abraham M. J., Murtola T., Schulz R., Páll S., Smith J. C., Hess B., Lindahl E. (2015). GROMACS: High performance molecular simulations through multi-level parallelism from laptops to supercomputers. SoftwareX.

[cit37] Parez S., Předota M., Machesky M. (2014). Dielectric Properties of Water at Rutile and Graphite Surfaces: Effect of Molecular Structure. J. Phys. Chem. C.

[cit38] Gavish N., Promislow K. (2016). Dependence of the dielectric constant of electrolyte solutions on ionic concentration: A microfield approach. Phys. Rev. E.

[cit39] Biriukov D., Fibich P., Předota M. (2020). Zeta Potential Determination from Molecular Simulations. J. Phys. Chem. C.

[cit40] Předota M., Machesky M. L., Wesolowski D. J. (2016). Molecular Origins of the Zeta Potential. Langmuir.

[cit41] Ohshima H. , Theory of Colloid and Interfacial Electric Phenomena, Elsevier, Academic Press, Amsterdam, 2006, vol. 12

[cit42] Döpke M. F., Hartkamp R. (2021). The importance of specifically adsorbed ions for electrokinetic phenomena: Bridging the gap between experiments and MD simulations. J. Chem. Phys..

[cit43] Brown M. A., Abbas Z., Kleibert A., Green R. G., Goel A., May S., Squires T. M. (2016). Determination of Surface Potential and Electrical Double-Layer Structure at the Aqueous Electrolyte-Nanoparticle Interface. Phys. Rev. X.

[cit44] Ma E., Ohno P. E., Kim J., Liu Y., Lozier E. H., Miller T. F., Wang H.-F., Geiger F. M. (2021). A New Imaginary Term in the Second-Order Nonlinear Susceptibility from Charged Interfaces. J. Phys. Chem. Lett..

[cit45] Diot J. L., Joseph J., Martin J. R., Clechet P. (1985). pH dependence of the Si/SiO_2_ interface state density for EOS systems: Quasi-static and AC conductance methods. J. Electroanal. Chem..

[cit46] Siu W. M., Cobbold R. S. C. (1979). Basic properties of the electrolyte—SiO_2_—Si system: Physical and theoretical aspects. IEEE Trans. Electron Devices.

[cit47] Söderström J., Ottosson N., Pokapanich W., Öhrwall G., Björneholm O. (2011). Functionalized nanoparticles in aqueous surroundings probed by X-ray photoelectron spectroscopy. J. Electron Spectrosc. Relat. Phenom..

[cit48] Sonnefeld J. (1996). Determination of Surface Charge Density Constants for Spherical Silica Particles Using a Linear Transformation. J. Colloid Interface Sci..

[cit49] Yamanaka J., Hayashi Y., Ise N., Yamaguchi T. (1997). Control of the surface charge density of colloidal silica by sodium hydroxide in salt-free and low-salt dispersions. Phys. Rev. E.

[cit50] Jena K. C., Covert P. A., Hore D. K. (2011). The Effect of Salt on the Water Structure at a Charged Solid Surface: Differentiating Second- and Third-order Nonlinear Contributions. J. Phys. Chem. Lett..

[cit51] Pezzotti S., Galimberti D. R., Gaigeot M.-P. (2019). Deconvolution of BIL-SFG and DL-SFG spectroscopic signals reveals order/disorder of water at the elusive aqueous silica interface. Phys. Chem. Chem. Phys..

[cit52] Chen S.-H., Singer S. J. (2019). Molecular Dynamics Study of the Electric Double Layer and Nonlinear Spectroscopy at the Amorphous Silica–Water Interface. J. Phys. Chem. B.

[cit53] Joutsuka T., Morita A. (2018). Electrolyte and Temperature Effects on Third-Order Susceptibility in Sum-Frequency Generation Spectroscopy of Aqueous Salt Solutions. J. Phys. Chem. C.

[cit54] Joutsuka T., Hirano T., Sprik M., Morita A. (2018). Effects of third-order susceptibility in sum frequency generation spectra: a molecular dynamics study in liquid water. Phys. Chem. Chem. Phys..

[cit55] Cai C., Azam Md. S., Hore D. K. (2021). Determining the Surface Potential of Charged Aqueous Interfaces Using Nonlinear Optical Methods. J. Phys. Chem. C.

[cit56] Ohno P. E., Chang H., Spencer A. P., Liu Y., Boamah M. D., Wang H., Geiger F. M. (2019). Beyond the Gouy–Chapman Model with Heterodyne-Detected Second Harmonic Generation. J. Phys. Chem. Lett..

[cit57] Wang H., Hu X.-H., Wang H.-F. (2021). Charge-Induced *χ*^(3)^ Susceptibility in Interfacial Nonlinear Optical Spectroscopy Beyond the Bulk Aqueous Contributions: The Case for Silica/Water
Interface. J. Phys. Chem. C.

[cit58] Barisik M., Atalay S., Beskok A., Qian S. (2014). Size Dependent Surface Charge Properties of Silica Nanoparticles. J. Phys. Chem. C.

