# Supplementary material for: Evolution of the electrical double layer with electrolyte concentration probed by second harmonic scattering
Source: Faraday Discuss. 2023 Jul 17;246:407–25. doi: 10.1039/d3fd00036b (PMC10568258; doi:10.1039/d3fd00036b)
Supplement: FD-246-D3FD00036B-s001 [file FD-246-D3FD00036B-s001.pdf]

Electronic Supplementary Material (ESI) for Faraday Discussions.
This journal is © The Royal Society of Chemistry 2023

Supplementary Information for:

Evolution of the Electrical Double Layer with Electrolyte Concentration Probed by Second Harmonic Scattering

Bingxin Chu[1], Denys Biriukov[2], Marie Bischoff[1, §], Milan Předota[3], Sylvie Roke[1] and Arianna Marchioro[1*]

[1]*Laboratory for fundamental BioPhotonics (LBP), Institute of Bioengineering (IBI), and Institute of Materials Science (IMX), School of Engineering (STI), École polytechnique fédérale de Lausanne (EPFL), CH-1015 Lausanne, Switzerland.*

[2]*Institute of Organic Chemistry and Biochemistry, Czech Academy of Sciences, Flemingovo nám. 2, 16610 Prague 6, Czech Republic*

[3]*Department of Physics, Faculty of Science, University of South Bohemia, Branišovská 1760, 370 05 České Budějovice, Czech Republic*

[§]*Present address: Oryl Photonics SA, c/o ZenPME Group Sàrl, Avenue Sevelin 28, CH-1004 Lausanne, Switzerland.*

**E-mail : arianna.marchioro@epfl.ch*

S1. Experimental parameters used for fitting the AR-SHS patterns

Table S1: Parameters used to fit the normalized second harmonic scattering patterns with the AR-SHS model. These parameters are common to all sets of SiO_2 data presented and fitted.

Second harmonic wavelength λ [nm]	516
Refractive index n_{H_2O} (@ 516nm)	1.33
Refractive index n_{SiO_2} (@ 516nm)	1.46
Temperature [°C]	23

Table S2-S4: Parameters used for fitting the normalized second harmonic scattering patterns of different diameter amorphous SiO_2 particles as a function of NaCl concentration (Figure S1) using the AR-SHS model. The radius represents the hydrodynamic radius measured by dynamic light scattering. The effective ionic strength is determined via conductivity as described in the Materials and Methods section. It includes the contribution from the added salt as well as the initial ionic strength of the solution of washed particles. For samples with no ions added intentionally, the ionic strength fitting parameter was left open, as indicated by (OS) corresponding to "open salt". The number of particles was calculated based on the sample mass and size.

Table S2: Parameters used for fitting the normalized second harmonic scattering patterns of 100 nm amorphous SiO_2 particles as a function of NaCl concentration.

Added ionic strength [μM]	0	50	100	250	500
Radius [nm]	66	62	62	60	59
Effective ionic strength [μM]	16 (OS)	62	111	263	515
Number of particles [/ml]	$2.3 \cdot 10^{11}$				

Table S3: Parameters used for fitting the normalized second harmonic scattering patterns of 200 nm amorphous SiO_2 particles as a function of NaCl concentration.

Added ionic strength [μM]	0	50	100	250	500	750	1000
Radius [nm]	90	88	87	86	85	84	85
Effective ionic strength [μM]	14 (OS)	61	111	266	528	775	1130
Number of particles [/ml]	$1.3 \cdot 10^{11}$						

Table S4: Parameters used for fitting the normalized second harmonic scattering patterns of 300 nm amorphous SiO_2 particles as a function of NaCl concentration.

Added ionic strength [μM]	0	50	100	250	500	750	1000	2500	5000
Radius [nm]	160	158	149	147	148	144	145	144	147
Effective ionic strength [μM]	10	57	102	286	484	718	974	2350	4690
Number of particles [/ml]	$3.3 \cdot 10^{10}$								

S2. AR-SHS patterns of 100, 200, and 300 nm diameter SiO_2 particles in NaCl solutions

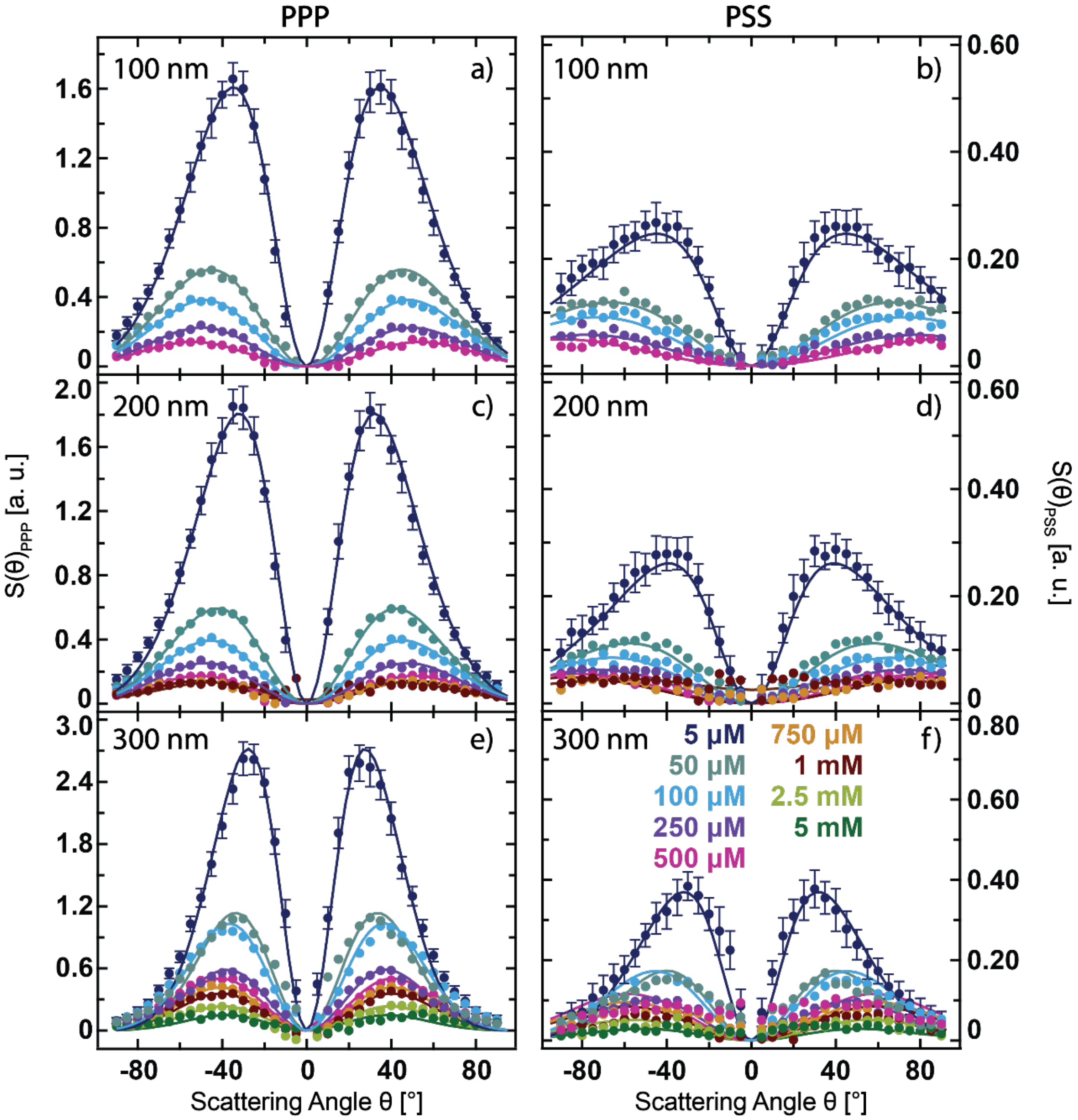

Figure S1: Measured AR-SHS patterns of SiO_2 in PPP and PSS polarization combination in NaCl solutions as a function of ionic strength. Patterns for three sizes are measured: 100 (a-b), 200 (c-d), and 300 nm (e-f). All the samples have the same total surface area (see Materials and Methods). Plain data points of different colors represent different ionic strengths of the aqueous environment. Solid lines represent the fits to the corresponding data points using the AR-SHS model detailed in Refs. 16, 22-24 of the main text. The summary of all parameters used for the fits can be found in tables S1 to S4.

S3. Molecular dynamics simulations

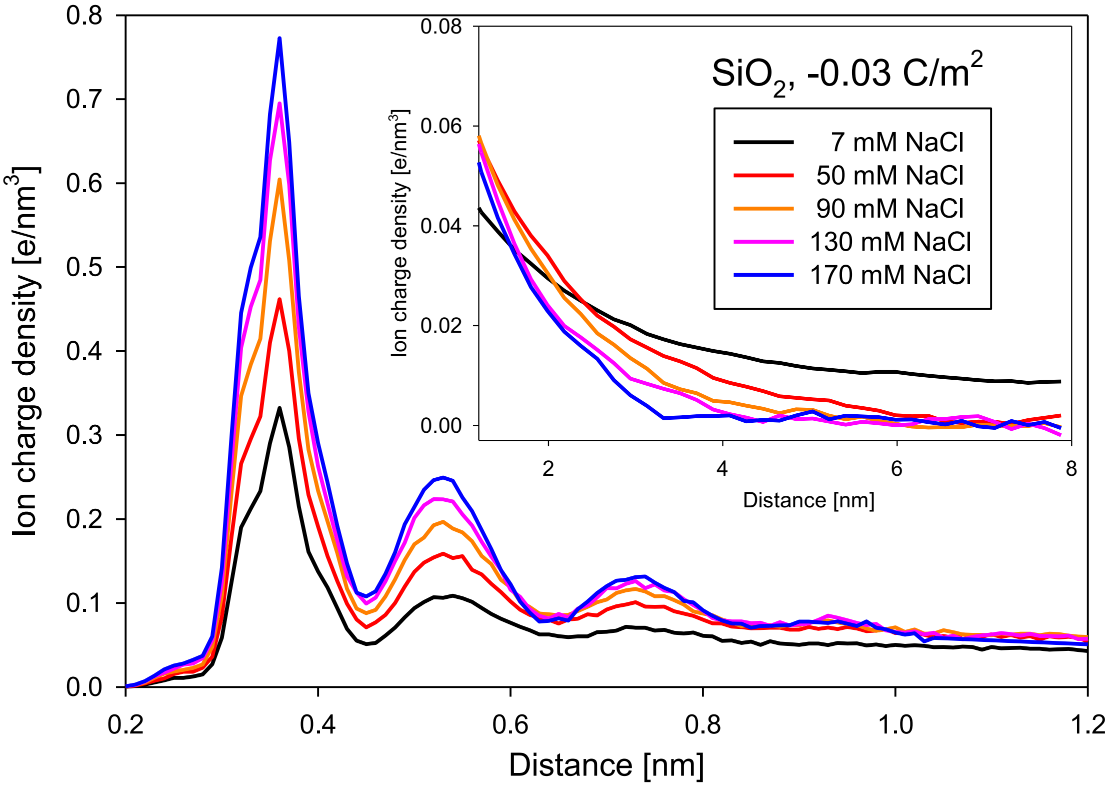

Figure S2: Difference between axial densities of Na^+ and Cl^-, yielding axial density of ion charge. Positive values indicated stronger screening of the negative surface charge. Since all systems are charge-neutral and the same surface charge -0.03 C/m^2 needs to be compensated, the integral of all these curves must be equal. It is evident that for higher bulk concentration of NaCl the surface charge is compensated at shorter distances, while for low bulk concentrations the effect of the surface charges extends to larger distances. The inset shows the behavior at larger distances.

S4. Zeta potential for 300 nm diameter SiO_2 particles in NaCl solutions

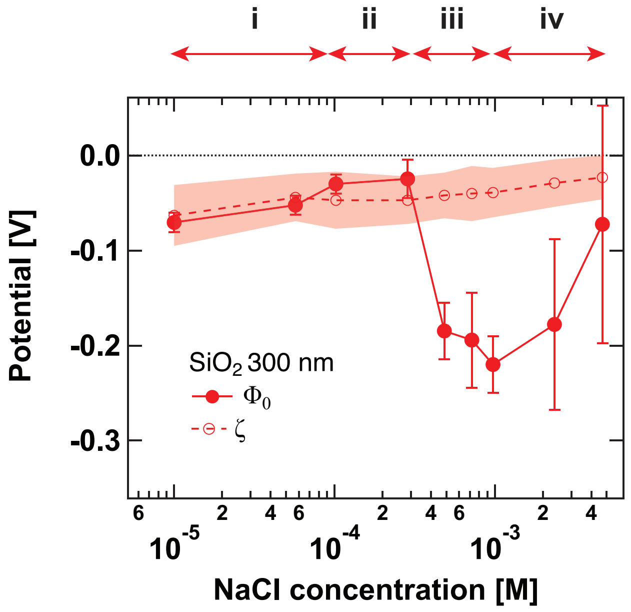

Figure S3: Surface potential Φ_0(red plain circles) and zeta potential ζ (red open circles) for 300 nm-diameter amorphous SiO_2 particles as a function of NaCl concentration. Errors bars for Φ_0 are indicated as red bars, while for ζ they are indicated as the colored red area (plain). While ζ and Φ_0 have similar trends in region (i) and (ii), it is evident from the graph that they differ significantly in region (iii) and (iv), suggesting that both quantities are not sensitive to the same phenomena.
